# Anemia and health-related quality of life in South Korea: data from the Korean national health and nutrition examination survey 2008–2016

**DOI:** 10.1186/s12889-019-6930-y

**Published:** 2019-06-13

**Authors:** Young-Ju Kim, Kyung Do Han, Kyung-Hwan Cho, Yang-Hyun Kim, Yong-Gyu Park

**Affiliations:** 1 Department of Medical Lifescience, The Catholic University College of Medicine, 222, Banpo-daero, Seocho-gu, Seoul 137-701 South Korea; 2 Department of Medical Statistics, The Catholic University College of Medicine, 222, Banpo-daero, Seocho-gu, Seoul 137-701 South Korea; 3 Department of Family Medicine, Korea University College of Medicine, 73 Inchon-ro, Seoungbuk-Gu, Seoul 136-705 South Korea

**Keywords:** Anemia, Quality of life, EuroQol five-dimensional questionnaire, Korean national health and nutrition examination survey

## Abstract

**Background:**

Anemia is associated with impaired quality of life (QoL). We examined the relationship between anemia and QoL in the Korean population using the EuroQol five-dimensional (EQ-5D) questionnaire.

**Methods:**

Data of 30,526 subjects were included from the Korean National Health and Nutrition Examination Survey (2008–2016). The QoL was assessed using three-levels of the EQ-5D questionnaire (G1, G2, and G3). Analysis of variance was used to compare the prevalence of anemia according to the three levels of health status in each of the five dimensions of EQ-5D. Multiple linear regression analysis was used to evaluate the association between hemoglobin level and QoL, and multivariable logistic regression analysis was used to evaluate the odds ratios (ORs) and 95% confidence intervals (CIs) for low levels of each of the five dimensions of EQ-5D.

**Results:**

As the level of EQ-5D was worse (from G1 to G3), the prevalence of anemia increased (p for trend < 0.001). Hemoglobin level and EQ-5D showed positive association after adjusting for all covariates such as age, sex, smoking, alcohol drinking, exercise, education, income, marital status, urban living, diabetes mellitus, hypertension, hypercholesterolemia, chronic kidney disease, total calorie intake, and protein intake. Subjects with anemia had increased ORs for low levels (G2 + G3) of each dimension of EQ-5D compared to subjects without anemia. ORs and 95% CIs for mobility, self-care, and usual activities were 1.208(1.078, 1.353), 1.161(0.98, 1.376), and 1.331(1.173, 1.51), respectively, after adjusting for all covariates. Pain/discomfort and anxiety/depression were not associated with increased ORs for low levels of EQ-5D.

**Conclusions:**

In South Korea, low QoL was associated with anemia, particularly in the mobility, self-care, and usual activities dimensions of EQ-5D.

**Electronic supplementary material:**

The online version of this article (10.1186/s12889-019-6930-y) contains supplementary material, which is available to authorized users.

## Background

Anemia is characterized by a decreased number and altered morphology of red blood cells. The World Health Organization (WHO) defines anemia as a hemoglobin (Hb) level below 13 g/dL in men and 12 g/dL in women [1]. With advances in preventive medicine and improved nutrition, the global prevalence of anemia has decreased from 40.2% in 1990 to 32.9% in 2010 [2]; in the United States (US), the prevalence of anemia was 6.5% in 2003–2013 [3]. In South Korea, data from the Korea National Health and Nutrition Examination Survey (KNHANES) revealed that the overall prevalence of anemia in individuals over 10 years of age was 8.2% in 2005–2015, and that it has decreased from 8.5% in 2005 to 6.4% in 2015 [4].

Although the world has made progress in reducing mortality and extending life expectancy over the past few decades [5, 6], people recognize that optimal health-related quality of life (HRQoL) is more important than just a life extension [5, 7]. Anemia is associated with morbidity and mortality [8, 9], and is the major cause of years lived with disability since 1990 [5], indicating that anemia often affects individuals with poor health who require comprehensive health care services [7]. In some studies, Hb ≤ 12 g/dL was associated with decreased QoL and functional status among patients with cancer [10, 11]. Anemia impairs HRQoL by causing a variety of symptoms from mild headache to cognitive impairment [3, 12]. Frequent experience of anemia symptoms may have a negative impact on daily life [8, 13–15]. Many studies on the relationship between anemia and QoL used relatively small samples or specific populations such as the elderly [7, 14, 15], patients with cancer [10, 11, 13, 16], and middle-aged women [17]. Therefore, we investigated the relationship between anemia and QoL using nationally representative data from the KNHANES.

## Methods

### Data source and subjects

We used data from the KNHANES 2008–2016. The KNHANES comprises a health examination, a health interview survey, and a nutritional survey, which are carried out by trained investigation team members (examiners and interviewers). The technical investigation team comprises a nurse, a nutritionist, and a health science major, whose investigative performance is regularly verified and maintained through training and field quality control to ensure consistent and reliable performance and reduce bias in the examinations and interviews. A nationwide survey has been conducted by the Division of Chronic Disease Surveillance at the Korean Ministry of Health and Welfare to monitor the general health and nutritional status of the South Korea population. The survey sampling is designed through stratified, multistage probability with proportional allocation based on age, sex, and geographic area from the 2005 National Census Registry, to represent the entire population in South Korea. Data were obtained through “confirmation of plan for raw data use” from the Korean Centers for Disease Control and Prevention through the KNHANES website (http://knhanes.cdc.go.kr) after submitting permission. The KNHANES was approved by the Institutional Review Board of the Korea Centers for Disease Control (IRB No. 1401–047-547), and all participants signed an informed consent form. This study meets the Helsinki Declaration-based ethical principles for medical research involving human subjects. Initially, the KNHANES 2008–2016 was completed by 76,909 subjects. We excluded 17,823 subjects who were under 19 years old and 8712 subjects with missing data. Thus, data from a total of 50,374 subjects were analyzed in this study.

### Measurements and definition of variables

#### Definition of anemia

The definition of anemia was based on WHO criteria for serum Hb level, which are < 13.0 g/dL in men, < 12.0 g/dL in non-pregnant women, and < 11.0 g/dL in pregnant women [1].

#### Sociodemographic variables

Information on age, sex, household income, and educational level was collected. Household income was classified into two groups, with the baseline set at the lowest quartile: a monthly income lower or greater than $1092.4. Educational level was classified into two groups, with a baseline of learning years of 0–9 or longer. Information on marital status (living with spouse/living without a spouse) and residential area (urban/rural) was also collected.

#### General health behaviors

General health behaviors such as current smoking status, alcohol consumption, and physical exercise were collected by self-report questionnaire. Subjects were asked about current smoking status and alcohol consumption during the current year. Smoking status was classified into two categories: current smoker or former smoker/never smoker. Alcohol consumption status was classified into two categories: alcohol drinker (glasses per day in a current month) or non-drinker. Physical exercise was classified into two categories based on a modified form of the International Physical Activity Questionnaire for Koreans [18]: regular walking or non-regular walking. Regular walking was considered when subjects performed walking more than 5 times a week for over 30 min per session. Information on the perceived level of stress (high or low) was obtained from the interviews. Daily intakes of total energy, fat, and protein were calculated based on the food items consumed using the 24-h recall method.

#### Anthropometric measurements

Body weight (kg) and height (cm) were measured with the subject wearing light clothing with no shoes. Waist circumference was measured at the midlevel between the end area of the rib cage and the iliac crest. Blood pressure was measured with a standard mercury sphygmomanometer (Baumanometer; W.A. Baum Co., Inc., Copiague, NY). Systolic and diastolic blood pressure was measured twice at 5-min intervals, and the average value was calculated.

#### Biochemical measurements

We checked blood urea nitrogen (BUN) (mg/dL), creatinine (mg/dL), fasting plasma glucose (mg/dL), high-density lipoprotein (HDL) cholesterol (mg/dL), triglycerides (mg/dL), Hb (g/dL), glycated Hb A1c (HbA1c) (%), ferritin (ng/mL), and vitamin D (IU). Blood samples were collected from the antecubital vein of each subject after fasting for > 8 h, and the blood samples were analyzed within 24 h of transportation. BUN, creatinine, glucose, HDL cholesterol, and triglycerides were measured by an automatic analyzer 7600 (Hitachi, Tokyo, Japan). Hb level was determined using the sodium lauryl sulfate Hb method measured by XE-2100D (Sysmex, Tokyo, Japan). HbA1c level was determined using high-performance liquid chromatography (HLC-723 G7; Tosoh, Tokyo, Japan). Ferritin level was determined using the immunoradiometric assay method. Vitamin D level was determined using the radioimmunoassay method and a 1470 WIZARD gamma counter (PerkinElmer).

#### Definition of chronic diseases

Chronic disease was defined by a doctor’s diagnosis or treatment history for the following diseases: cardiovascular disease (including angina pectoris, myocardial infarction, and stroke), chronic kidney disease, diabetes mellitus (DM), hypercholesterolemia, and hypertension (HTN). Chronic kidney disease (CKD) was defined as an estimated glomerular filtration rate (eGFR) calculated using the Modification of Diet in Renal Disease equation < 60 mL/min/1.73 m^2^ [19].

#### Measurement of HRQoL

The EQ-5D questionnaire has been preselected for the measurement of a subject’s HRQoL in South Korea, and the validation of the Korean version has been reported previously [20]. The EQ-5D questionnaire includes a simple self-report section that corresponds to five dimensions of mobility, self-care, usual activity, pain/discomfort, and anxiety/depression. The EQ-5D scores were calculated using the weighted model to transform these health statuses into South Korea population-based health statuses [21]. The five questions regarding health status were surveyed and we used the EQ-5D calculation formula using their estimated weighted quality value for Koreans by Nam et al. [22]. The formula is: EQ-5D index = 1-(0.05 + 0.096 × M2 + 0.418 × M3 + 0.046×SC2+0.136 × SC3 + 0.051 × UA2 + 0.208 × UA3 + 0.037 × PD2 + 0.151 × PD3 + 0.043 × AD2 + 0.158 × AD3 + 0.05 × N3). [M2-Mobility “level 2” = 1; otherwise, 0; M3 - Mobility “level 3” = 1; otherwise, 0; SC2 - Self-care “level 2” = 1; otherwise, 0; SC3 - Self-care “level 3” = 1; otherwise, 0; UA2 - Usual activities “level 2” = 1; otherwise, 0; UA3 - Usual activities “level 3” = 1; otherwise, 0; PD2 - Pain/discomfort “level 2” = 1; otherwise, 0; PD3 - Pain/ discomfort “level 3” = 1; otherwise, 0; AD2-Anxiety/depression “level 2” = 1; otherwise, 0; AD3-Anxiety/depression “level 3” = 1; otherwise, 0; N3 - Only one “level 3” = 1, and the rest = 0]. Higher EQ-5D scores indicate better HRQoL. We used the three-level version of EuroQol five-dimensional (EQ-5D) questionnaire to evaluate HRQoL (G1, no problems; G2, moderate problems; and G3, serious problems). We defined a high level of HRQoL as G1 and a low level of HRQoL as G2 + G3.

### Statistical analysis

Statistical analyses were conducted using survey procedures from SAS software version 9.2 (SAS Institute Inc., Cary, NC) to account for the complex sampling design and to estimate nationally representative prevalence rates. The data are presented as means ± standard errors (SE) for continuous variables and as percentages (SE) for categorical variables. The chi-square test for categorical variables or the student independent t-test for continuous variables was conducted to assess the differences in characteristics between the non-anemia group and the anemia group. Analysis of variance was used to compare the prevalence of anemia according to the three levels of health status (G1, G2, and G3) of each of the five dimensions of EQ-5D. Multiple linear regression analysis was used to evaluate the association between Hb level and HRQoL after adjusting for covariates. Model 1 was adjusted for age and sex. Model 2 adjusted for age, sex, current smoking status, alcohol consumption, physical exercise, income, education level, and spouse. Model 3 was adjusted for the variables included in model 2 with the addition of DM, HTN, hypercholesterolemia, CKD, total calorie intake, and protein intake. Multiple logistic regression analysis was used to evaluate the odds ratios (ORs) and 95% confidence intervals (CIs) for low levels (G2 + G3) of each of the five dimensions of EQ-5D using non-anemia subjects as the reference group. All *p*-values of less than 0.05 were considered statistically significant.

## Results

Table 1 shows the baseline characteristics of the participants according to presence of anemia. Subjects with anemia had higher Hb and ferritin levels (both *p* < 0.001). Subjects with anemia were older and had lower protein intake, and there were lower percentage of men, smokers, alcohol drinkers, subjects with an education ≥9 years, subjects who lived in urban areas, subjects with hypercholesterolemia; however, there was a higher percentage of subjects with low income (Q1), who lived with their spouse, and had DM or CKD (all *p* < 0.001). Weight, body mass index, waist circumference, and total calorie intake were lower in subjects with anemia than in subjects without anemia (all *p* < 0.05). EQ-5D was also lower in subjects with anemia than in subjects without anemia (0.95 vs 0.93, respectively and *p* < 0.001).

**Table 1:** Patient characteristics according to anemia status

	Non-anemia (*n* = 45,827)	Anemia (*n* = 4547)	*p*-value
Age (years)	44.86 ± 0.13	48.92 ± 0.34	< 0.001
Men (yes, %)	52.57(0.24)	18.02(0.72)	< 0.001
Height (cm)	164.68 ± 0.06	159.11 ± 0.13	< 0.001
Weight (kg)	64.86 ± 0.08	57.61 ± 0.18	< 0.001
Body mass index (kg/m^2^)	23.82 ± 0.02	22.74 ± 0.06	< 0.001
Waist circumference (cm)	81.61 ± 0.08	77.65 ± 0.18	< 0.001
Hemoglobin (g/dL)	14.45 ± 0.01	11.09 ± 0.02	< 0.001
BUN (mg/dL)	14 ± 0.03	14.74 ± 0.16	<.0001
Creatinine (mg/dL)	0.85 ± 0	0.85 ± 0.02	0.846
eGFR (mL/min/1.73 m^2^)	95.61 ± 0.16	94.61 ± 0.48	0.033
Glucose (mg/dL)	97.67 ± 0.14	96.6 ± 0.41	0.012
Ferritin^a^ (mg/dL)	61.41(60.48–62.35)	14.72(13.76–15.74)	< 0.001
Current smoking (yes, %)	24.22(0.28)	7.03(0.46)	< 0.001
Alcohol drinking (yes, %)	60.87(0.31)	42.45(0.92)	< 0.001
Regular walking (yes, %)	41.14(0.33)	39.52(0.87)	0.075
Income (Q1) (yes, %)	14.6(0.31)	20.91(0.74)	< 0.001
Education > 9 years (yes, %)	73.76(0.36)	66.77(0.88)	< 0.001
Urban living (yes, %)	82.45(0.86)	80.28(1.17)	0.005
Spouse (yes, %)	76.96(0.35)	86.43(0.74)	< 0.001
DM (yes, %)	8.61(0.16)	11.93(0.58)	< 0.001
HTN (yes, %)	25.85(0.29)	26.05(0.78)	0.798
Hypercholesterolemia (yes, %)	13.52(0.2)	10.23(0.51)	< 0.001
CKD (yes, %)	1.53(0.06)	8.67(0.48)	< 0.001
EQ-5D (mean)	0.95 ± 0.06	0.93 ± 0.24	< 0.001
Total calorie intake (cal/day)	2069.97 ± 7.06	1721.91 ± 14.64	< 0.001
Protein intake (%)	14.78 ± 0.03	14.02 ± 0.08	< 0.001

^a^ Geometric means (95% confidence intervals)

Data are presented as means ± standard errors (SE) or percentages (SE)

The p-values were obtained by chi-square test or student’s t-test

*BUN* blood urea nitrogen, *eGFR* estimated glomerular filtration rate, *DM* diabetes mellitus, *HTN* hypertension, *CKD* chronic kidney disease, *EQ-5D* EuroQol five-dimensional questionnaire

**Fig. 1:**
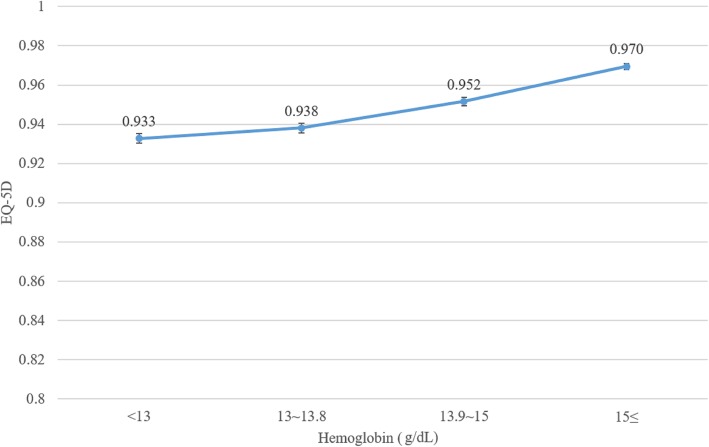
EQ-5D score according to quartile of hemoglobin levels. EQ-5D: EuroQol five-dimensional questionnaire

Figure 1 shows the EQ-5D score according to the quartile of Hb levels. As the Hb levels increase, EQ-5D also increase from 0.933 to 0.970.

Table 2 shows the results of multiple linear regression analysis between Hb level and quality of life. As Hb level increases per 1 g/dL, the EQ-5D positively increases after adjusting for all covariates in model 3 (the estimate = 0.0016 ± 0.0005 and *p* < 0.001). Multiple linear regression analysis between other variables and EQ-5D is shown in Additional file 1: Table S1.

**Table 2:** Multiple linear regression analysis of EQ-5D index score by increase of hemoglobin (1 g/dL)

Hemoglobin (per 1 g/dL)	EQ-5D	
	Estimate (SE)	*p-*value
Model 1	0.0012 ± 0.0005	0.017
Model 2	0.0015 ± 0.0005	0.001
Model 3	0.0016 ± 0.0005	< 0.001

Model 1: adjusted for age and sex

Model 2: adjusted for age, sex, smoking, alcohol drinking, exercise, education, income, and marital status

Model 3: adjusted for age, sex, smoking, alcohol drinking, exercise, education, income, marital status, urban living, diabetes mellitus, hypertension, hypercholesterolemia, chronic kidney disease, total calorie intake, and protein intake

*EQ-5D* EuroQol five-dimensional questionnaire

**Fig. 2:**
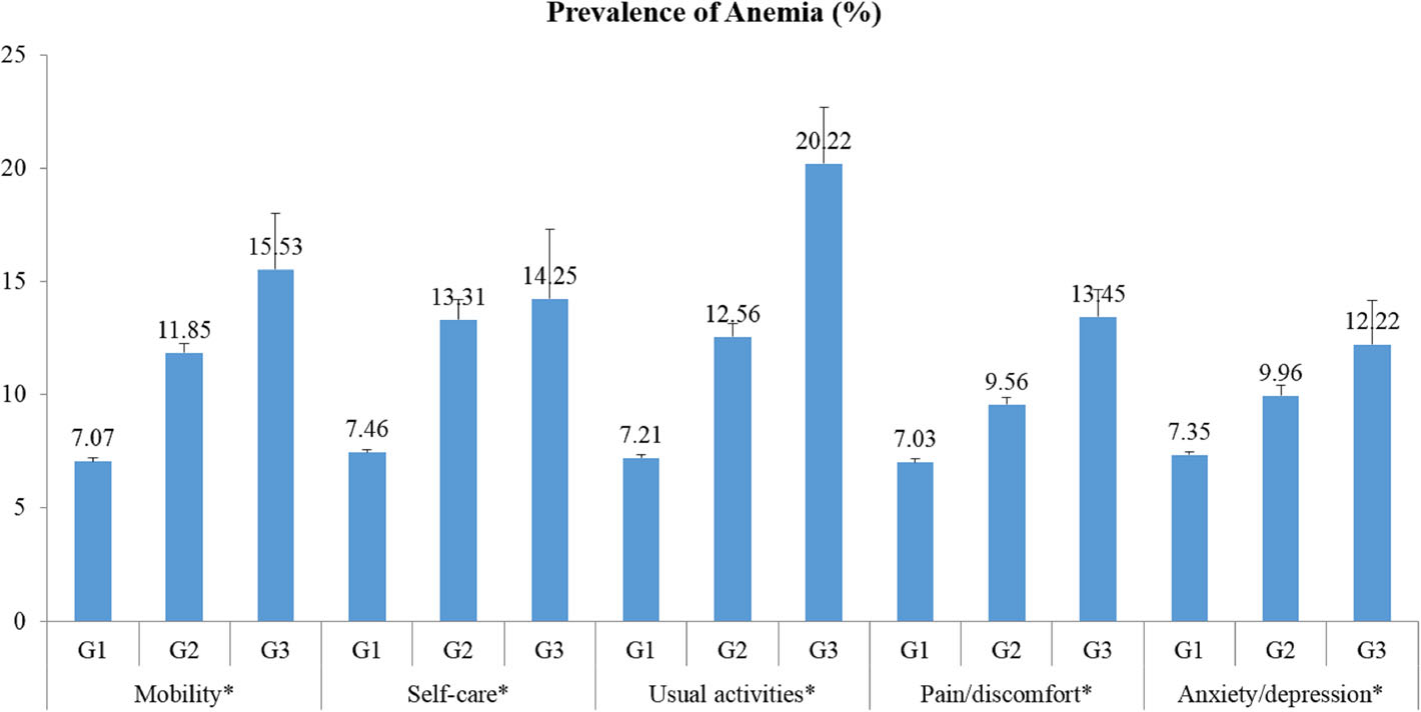
Prevalence of anemia according to the three levels of each of the five EQ-5D Components. G1: no problems, G2: some problems, and G3: unable to perform or extreme problems. Data were analyzed by the linear trend test. Bars mean standard errors. * *p* < 0.001 and p for trend < 0.001 EQ-5D: EuroQol five-dimensional questionnaire

Figure 2 shows the prevalence of anemia according to the three levels of the five EQ-5D dimensions. As the level was changed from G1 to G3, the prevalence of anemia increased (all p for trend < 0.001), and there were significant differences among the three levels of each of the five dimensions of EQ-5D (all *p* < 0.001). The highest prevalence of anemia was found in the G3 level of each of the five EQ-5D dimensions in the following order (20.20% in usual activities, 15.53% in mobility, 14.25% in self-care, 13.45% in pain/discomfort, and 12.22% in anxiety/depression).

Multivariate logistic regression analysis was done in Table 3 to calculate the OR for low levels (G2 + G3) of each dimension of EQ-5D compared to subjects without anemia. Subjects with anemia had increased ORs for low levels of each dimension of EQ-5D in mobility, self-care, and usual activities (OR and 95% CI = 1.208(1.078, 1.353), 1.161(0.98, 1.376), and 1.331(1.173, 1.51)) after adjusting for all covariates. However, pain/discomfort and anxiety/depression were not associated with increased ORs for low levels of EQ-5D (Table 3). Multiple logistic regression analysis for the OR for low levels (G2 + G3) of each dimension of EQ-5D in other variables is shown in Additional file 1: Table S2.

**Table 3:** Multivariate adjusted odds ratios for low levels of each component of EQ-5D

Components	Model 1		Model 2		Model 3	
	OR (95% CI)	*p*-value	OR (95% CI)	*p*-value	OR (95% CI)	*p*-value
Mobility	1.198(1.085,1.323)	< 0.001	1.211(1.089,1.347)	< 0.001	1.208(1.078,1.353)	0.001
Self-care	1.223(1.045,1.432)	0.012	1.211(1.026,1.43)	0.024	1.161(0.98,1.376)	0.084
Usual activities	1.349(1.206,1.508)	< 0.001	1.353(1.2,1.526)	< 0.001	1.331(1.173,1.51)	< 0.001
Pain/discomfort	1.097(1.01,1.191)	0.028	1.077(0.989,1.174)	0.09	1.07(0.977,1.171)	0.144
Anxiety/depression	1.069(0.96,1.189)	0.225	1.069(0.956,1.196)	0.24	1.063(0.943,1.199)	0.314

Model 1: adjusted for age and sex

Model 2: adjusted for age, sex, smoking, alcohol drinking, exercise, education, income, and marital status

Model 3: adjusted for age, sex, smoking, alcohol drinking, exercise, education, income, marital status, urban living, diabetes mellitus, hypertension, hypercholesterolemia, chronic kidney disease, total calorie intake, and protein intake

*OR* odds ratio, *CI* confidence interval, *EQ-5D* EuroQol five-dimensional questionnaire

## Discussion

In this study, anemia was more prevalent in subjects with low level of health status in each of the five dimensions of EQ-5D, and subjects with anemia had increased ORs for low EQ-5D, especially for mobility, self-care, and usual activities than subjects without anemia after adjusting for all covariates.

In this study, anemia was more prevalent among women and subjects with older age, poor nutritional status, lower education, and lower income level. Women of reproductive age (15–49 years) are known to be vulnerable to anemia worldwide [1]. The prevalence of anemia in US women aged 40–49 years is 10.7% [3]. The prevalence of anemia in Korean women aged 40–49 years was 16.1% in 2015, which is higher than in the US [4]. Firquet et al. reported that anemia in women in this age group may be caused by low iron intake, increased iron requirements, heavy menstrual bleeding, and malabsorption [17]. In particular, they reported that women of this age often restrict food intake or exercise excessively to lose weight [17]. In KNHANES 2008–2015, the rate of nutritional insufficiency among South Korean adults aged 30–49 years was 14.0% in women and 4.9% in men [4]. In general, poor income and lower education levels are known to be associated with reduced accessibility to medical services [23]. Therefore, subjects with anemia with lower level of socioeconomic status may not recognize their anemic status and may be left untreated [12, 13]. The symptoms of mild anemia in otherwise healthy adults are considered to be relatively not severe, yet they may continuously affect activities of daily living, especially in the elderly [7, 14, 15], due to the deterioration of overall body function [15, 24].

It has been reported that anemia affects QoL, especially physical performance, in the elderly and frail patients [7, 8, 13, 22, 25]. In the US, anemia is associated with mobility difficulty and lower mobility difficulty among women, after adjusting for chronic diseases [22]. Similarly, we found an association between anemia and HRQoL, namely in mobility, self-care, and usual activities. In the Korean Longitudinal Study on Health and Aging study, anemia was associated with activities of daily living in the elderly population, especially physical functioning and instrumental active daily living [7]. The nutrients ingested are synthesized as energy with the help of oxygen. However, Hb is decreased in subjects with anemia, and oxygen supply is reduced [3, 26]. This mechanism may explain why subjects with anemia have limited physical performance. Moreover, decline of muscle strength and muscle density is also associated with anemia [26], which results in the decline of mobility, self-care, and usual activities. Anemia is associated with chronic diseases such as cancer, CKD, and inflammatory bowel disease, which are commonly accompanied by iron deficiency, inflammation, and organ complications [8, 16]. These conditions may also cause pain. However, we did not find an association between anemia and pain in EQ-5D. Similarly, no significant relationship was found between anemia and QoL (pain) among Dutch patients with heart failure [27]. Inversely, anemia treatment could have a positive effect on most chronic diseases. It has been reported that the improvement of Hb levels is associated with QoL [8, 16, 17], and even a 1-g/dL increase in Hb from 11 g/dL to 12 g/dL resulted in an improvement of QoL [28]. In some studies, anemia and the level of anemia have been associated with depression and severity of depression [26, 29, 30], but our study did not show this relationship. Ethnic and cultural difference may be the reason for the different results, although low Hb level is associated with increased cerebral blood flow in frontal, hippocampal, and temporal regions, which are involved in depression pathways [31].

This study has some strengths. This is the first study using a large amount of nationwide data to examine the relationship between anemia and HRQoL. The association was evaluated by multiple logistic regression analyses after adjusting for various confounding factors. Moreover, we analyzed the association between anemia and HRQoL in each of the five dimensions of EQ-5D. However, our study has some limitations. First, a causal relationship between anemia and HRQoL could not be drawn from this study because of its cross-sectional design. Second, some health-related variables were not included as confounding factors (e.g., iron intake, supplement intake, or anemia-related inflammatory markers). Third, we only used EQ-5D to measure HRQoL, although there are many other tools for measuring HRQoL. Forth, we did not include people under 19, because different measurement tools were used to survey in subjects under 19 years old.

## Conclusions

Low HRQoL was associated with anemia in South Korean adults, namely in the EQ-5D dimensions of mobility, self-care, and usual activities. Physicians should consider HRQoL in subjects with anemia. Moreover, the government do their effort to improve nutritional status of anemia patients to improve the HRQOL and further prospective studies about improving of HRQOL by the treatment of anemia are needed.

## Additional file

Additional file 1: **Table S1.** Multiple linear regression analysis of EQ-5D index score and covariates. **Table S2.** Multivariable logistic regression analysis on the OR for low levels of each component of EQ-5D. (DOCX 20 kb)

## Abbreviations

BUN: Blood urea nitrogen
CI: Confidence intervals
CKD: Chronic kidney disease
DM: Diabetes mellitus
eGFR: Estimated glomerular filtration rate
EQ-5D: EuroQol five-dimensional
Hb: Hemoglobin
HbA1c: Glycated Hb A1c
HDL: High-density lipoprotein
HRQoL: Health-related quality of life
HTN: Hypertension
KNHANES: Korea National Health and Nutrition Examination Survey
OR: Odds ratio
SE: Standard errors
US: United States
WHO: World Health Organization
