# Supplementary material for: Anemia and health-related quality of life in South Korea: data from the Korean national health and nutrition examination survey 2008–2016
Source: BMC Public Health. 2019 Jun 13;19:735. doi: 10.1186/s12889-019-6930-y (PMC6567528; doi:10.1186/s12889-019-6930-y)
Supplement: Supplementary file 1 — Table S1. Multiple linear regression analysis of EQ-5D index score and covariates. Table S2. Multivariable logistic regression analysis on the OR for low levels of each component of EQ-5D. (DOCX 20 kb) [file 12889_2019_6930_MOESM1_ESM.docx]

Supplement table 1. Multiple linear regression analysis of EQ-5D index score and covariates

| Variables | EQ-5D | |
| --- | --- | --- |
|  | Estimate (SE) | p*-*value |
| Age (years) | -0.0013±0.0001 | <0.001 |
| Men (yes, %) | -0.0175±0.0016 | <0.001 |
| Current smoking (yes, %) | -0.009±0.0015 | <0.001 |
| Alcohol drinking (yes, %) | 0.0072±0.0011 | <0.001 |
| Regular walking (yes, %) | 0.0105±0.0010 | <0.001 |
| Income (Q1) (yes, %) | -0.0042±0.0022 | <0.001 |
| Education >9 years (yes, %) | 0.0317±0.0016 | <0.001 |
| Urban living (yes, %) | 0.0041±0.0016 | 0.011 |
| Spouse (yes, %) | -0.0207±0.0016 | <0.001 |
| DM (yes, %) | -0.0131±0.0025 | <0.001 |
| HTN (yes, %) | -0.0058±0.0014 | <0.001 |
| Hypercholesterolemia (yes, %) | -0.0061±0.0018 | 0.001 |
| CKD (yes, %) | -0.0315±0.0051 | <0.001 |
| Total calorie intake (kcal/day) | 0.0024±0.0006 | <0.001 |
| Protein intake (%) | 0.0005±0.0001 | <0.001 |

DM; diabetes mellitus, HTN; hypertension, CKD; chronic kidney disease, EQ-5D: EuroQol five-dimensional questionnaire

Supplement table 2. Multivariable logistic regression analysis on the OR for low levels of each component of EQ-5D

|  | OR (95% CI) | | | | | | | | | |
| --- | --- | --- | --- | --- | --- | --- | --- | --- | --- | --- |
| Variables | Mobility | p-value | Self-care | p-value | Usual activities | p-value | Pain/discomfort | p-value | Anxiety/depression | p-value |
| Hemoglobin (mg/dL) | 1.208(1.078,1.353) | 0.001 | 1.161(0.98,1.376) | 0.084 | 1.331(1.173,1.51) | <0.001 | 1.07(0.977,1.171) | 0.144 | 1.063(0.943,1.199) | 0.314 |
| Age (years) | 1.055(1.051,1.059) | <0.001 | 1.06(1.052,1.067) | <0.001 | 1.041(1.036,1.046) | <0.001 | 1.017(1.014,1.02) | <0.001 | 1.007(1.003,1.011) | 0.001 |
| Men (yes, %) | 1.564(1.422,1.721) | <0.001 | 1.05(0.897,1.229) | 0.547 | 1.327(1.18,1.492) | <0.001 | 1.657(1.544,1.777) | <0.001 | 2.143(1.925,2.386) | <0.001 |
| Current smoking (yes, %) | 1.37(1.212,1.548) | <0.001 | 1.279(1.066,1.535) | 0.008 | 1.259(1.097,1.444) | 0.001 | 1.144(1.047,1.25) | 0.003 | 1.285(1.138,1.451) | <0.001 |
| Alcohol drinking (yes, %) | 0.833(0.766,0.907) | <0.001 | 0.888(0.774,1.019) | 0.090 | 0.748(0.676,0.828) | <0.001 | 0.848(0.798,0.902) | <0.001 | 0.995(0.916,1.081) | 0.906 |
| Regular exercise (yes, %) | 0.726(0.672,0.784) | <0.001 | 0.706(0.623,0.802) | <0.001 | 0.794(0.725,0.868) | <0.001 | 0.861(0.814,0.911) | <0.001 | 0.838(0.772,0.908) | <0.001 |
| Income (Q1) (yes, %) | 1.781(1.631,1.946) | <0.001 | 1.917(1.654,2.223) | <0.001 | 2.131(1.921,2.363) | <0.001 | 1.511(1.402,1.628) | <0.001 | 1.852(1.68,2.042) | <0.001 |
| Education >9 years (yes, %) | 0.434(0.394,0.478) | <0.001 | 0.594(0.501,0.705) | <0.001 | 0.459(0.407,0.518) | <0.001 | 0.623(0.578,0.671) | <0.001 | 0.658(0.59,0.732) | <0.001 |
| Urban living (yes, %) | 0.863(0.788,0.945) | 0.001 | 0.811(0.704,0.936) | 0.004 | 0.896(0.805,0.998) | 0.047 | 0.928(0.859,1.002) | 0.057 | 1.074(0.967,1.192) | 0.184 |
| Spouse (yes, %) | 1.547(1.266,1.889) | <0.001 | 2.084(1.478,2.938) | <0.001 | 1.832(1.461,2.299) | <0.001 | 1.018(0.912,1.136) | 0.757 | 1.393(1.211,1.603) | <0.001 |
| DM (yes, %) | 1.327(1.2,1.468) | <0.001 | 1.664(1.443,1.919) | <0.001 | 1.311(1.17,1.47) | <0.001 | 1.149(1.054,1.253) | 0.002 | 1.159(1.031,1.302) | 0.014 |
| HTN (yes, %) | 1.258(1.164,1.359) | <0.001 | 1.152(1.007,1.319) | 0.039 | 1.191(1.08,1.313) | <0.001 | 1.011(0.946,1.081) | 0.738 | 0.988(0.899,1.086) | 0.803 |
| Hypercholesterolemia (yes, %) | 1.149(1.049,1.258) | 0.003 | 1.175(1.011,1.364) | 0.035 | 1.187(1.069,1.318) | 0.001 | 1.19(1.106,1.281) | <0.001 | 1.189(1.08,1.309) | <0.001 |
| CKD (yes, %) | 1.324(1.128,1.555) | <0.001 | 1.125(0.909,1.392) | 0.279 | 1.327(1.117,1.577) | 0.001 | 1.14(0.989,1.315) | 0.071 | 0.964(0.799,1.164) | 0.706 |
| Total calorie intake (kcal/day) | 0.896(0.84,0.956) | <0.001 | 0.82(0.738,0.912) | <0.001 | 0.863(0.801,0.929) | <0.001 | 1.013(0.974,1.054) | 0.522 | 0.96(0.907,1.016) | 0.154 |
| Protein intake (%) | 0.989(0.979,0.999) | 0.038 | 0.996(0.978,1.015) | 0.689 | 0.997(0.983,1.011) | 0.682 | 0.992(0.985,1) | 0.040 | 0.99(0.979,1) | 0.052 |

DM; diabetes mellitus, HTN; hypertension, CKD; chronic kidney disease, EQ-5D: EuroQol five-dimensional questionnaire, OR; odds ratio, CI; confidence interval
